# Incisional hernia and the risk of incident depression: a population-based propensity score–matched cohort study

**DOI:** 10.1007/s10029-026-03696-6

**Published:** 2026-05-07

**Authors:** Andreas Krieg, Sarah Krieg, Karel Kostev

**Affiliations:** 1 Department of General and Visceral Surgery, Thoracic Surgery and Proctology, University Hospital Herford, Medical Campus OWL, Ruhr University Bochum, Schwarzenmoorstr. 70, Herford, 32049 Germany; 2 University Clinic for People with Neurodevelopmental Disorders, Mara Hospital, Medical School and University Medical Center OWL, Bielefeld University, Bielefeld, 33617 Germany; 3 Epidemiology, IQVIA, Frankfurt, 60549 Germany

**Keywords:** Abdominal wall surgery, Mental health, Psychiatric comorbidity, Health-related quality of life, Population-based study, Real-world data

## Abstract

**Purpose:**

Incisional hernia is a common long-term complication of abdominal surgery and is traditionally seen as a structural defect. However, recent patient-centered research suggests that abdominal wall pathology may also impose substantial psychological burden. Whether incisional hernia is associated with an increased risk of clinically diagnosed depression at the population level remains unclear.

**Methods:**

This retrospective cohort study used data from the German Disease Analyzer database (IQVIA). Adults with a first documented diagnosis of incisional hernia (ICD-10: K43.0–K43.2) between 2005 and 2024 were identified. Individuals with recent psychiatric disorders were excluded to assess incident depression. Patients were matched 1:1 to controls without hernia using propensity scores based on age, sex, index year, consultation frequency, somatic comorbidities, and remote history of depression. The primary outcome was incident depression (ICD-10: F32, F33) within five years. Associations were analyzed using conditional Cox regression.

**Results:**

A total of 10,075 patients with incisional hernia were matched to 10,075 patients without hernia. During five years of follow-up, 18.4% of patients with and 16.5% without incisional hernia were diagnosed with depression. Incisional hernia was associated with a slightly increased risk of incident depression (hazard ratio 1.12; 95% confidence interval 1.04–1.20). The association was more pronounced among women and among individuals without prior depression.

**Conclusion:**

Incisional hernia is associated with a slightly increased risk of clinically diagnosed depression. These findings indicate a modest statistical association between incisional hernia and subsequent depression diagnoses in routine care. While the magnitude of the association was small, awareness of potential psychosocial comorbidity may be relevant in selected clinical contexts.

**Supplementary Information:**

The online version contains supplementary material available at 10.1007/s10029-026-03696-6.

## Introduction

 Incisional hernia remains one of the most common long-term complications following abdominal surgery. Although advances in minimally invasive approaches and randomized evidence supporting optimized midline closure strategies—particularly small-bites suturing—have reduced hernia formation in selected settings [1, 2], clinically relevant rates persist after midline laparotomy, especially among high-risk patients [3]. As surgical complexity increases and populations age, the absolute burden of incisional hernia remains substantial, maintaining its relevance as a major challenge in abdominal wall surgery. Given the increasing number of abdominal procedures performed worldwide, the absolute burden of incisional hernia continues to grow. Traditionally, incisional hernia has been conceptualized predominantly as a structural defect of the abdominal wall, and surgical research has largely focused on technical parameters such as mesh reinforcement, operative approach, recurrence rates, and postoperative complications [4–7].

Patient-centered outcomes, including quality of life (QoL) and psychosocial well-being, have historically received comparatively less attention [8]. However, accumulating evidence suggests that this purely anatomical perspective does not fully capture the patient experience. Health-related quality of life (HRQoL) has emerged as a critical outcome parameter in abdominal wall surgery. A systematic review by Grove et al. highlighted substantial heterogeneity in instruments used to assess QoL in patients with abdominal wall hernias and emphasized that psychosocial domains are often inconsistently captured [9]. This observation suggests that current surgical outcome metrics may not fully capture the aspects of disease burden that patients perceive as most relevant.

Recent qualitative investigations have provided important insight into this gap. In a patient-centered qualitative study, Smith and colleagues demonstrated that individuals living with abdominal wall hernia prioritize domains extending beyond physical symptoms, including mental health, body image, interpersonal relationships, and employment-related concerns when describing their disease burden [10]. Psychological distress and feelings of vulnerability emerged as recurrent themes within the mental health domain. In a subsequent investigation, the same group explicitly explored mental health aspects and reported that anxiety, low mood, and diminished confidence were commonly experienced by patients [11]. More recently, disturbances in body image were identified as a central and often underrecognized dimension of abdominal wall pathology, with patients describing embarrassment, altered body perception, and avoidance behaviors [12]. These findings suggest that abdominal wall hernia may represent not only a mechanical defect but also a sustained psychosocial stressor. Chronic pain, functional limitation, and visible abdominal contour changes may influence self-perception and social interaction. While improvements in HRQoL have been documented following incisional hernia repair in randomized and observational studies [13, 14], the extent to which hernia-associated psychological burden translates into clinically relevant psychiatric morbidity remains unclear.

Depression is a leading cause of disability worldwide and affects hundreds of millions of individuals globally [15]. It is strongly associated with chronic somatic conditions characterized by persistent symptoms, pain, and reduced functional capacity [16]. Furthermore, in surgical patients, depressive symptoms have been associated with impaired postoperative recovery, heightened pain perception, prolonged hospitalization, and reduced patient satisfaction [17–20]. Despite repeated qualitative descriptions of emotional distress among patients with abdominal wall hernia, there is a paucity of longitudinal epidemiological data examining whether incisional hernia is associated with an increased risk of incident depression.

Understanding this potential association is clinically relevant for several reasons. First, incisional hernia is common, and even modest increases in psychiatric risk may be relevant from a population-health perspective. Second, if psychological vulnerability is part of the disease trajectory, it may influence symptom perception, healthcare utilization, and outcomes after surgical repair. Third, recognition of mental health risk could inform patient counseling, shared decision-making, and the development of integrated care pathways.

To date, no large-scale population-based study has evaluated whether adults with newly diagnosed incisional hernia are at increased risk of developing clinically documented depression compared with individuals without hernia in routine care settings. Therefore, the aim of the present study was to investigate whether a newly documented diagnosis of incisional hernia is associated with an increased risk of subsequent clinically diagnosed depression in a representative outpatient cohort using propensity score matching and longitudinal follow-up.

Importantly, this study was not designed to evaluate the therapeutic impact of hernia repair or to assess psychological outcomes before and after surgical intervention. Rather, it addresses a population-level epidemiological question that has not previously been examined in a large longitudinal primary care dataset. By quantifying the psychiatric dimension of abdominal wall pathology, this analysis seeks to contribute complementary evidence to the existing literature focused on postoperative QoL outcomes.

## Methods

### Database

We conducted a retrospective cohort analysis using anonymized outpatient data from the German IQVIA Disease Analyzer database. This database compiles routinely documented diagnoses, prescriptions, and demographic information from electronic practice management systems of general and specialist practices throughout Germany. Approximately 3,000 practices contribute data, and sampling procedures are designed to reflect national distributions of physician specialty, region, and practice characteristics. Its sampling approach is based on statistics from the German Medical Association to ensure representativeness with respect to practice type, federal state, community size, and physician age. Previous validation studies have shown that the Disease Analyzer database is broadly representative of outpatient care in Germany with regard to physician characteristics and regional distribution [21]. The database has been widely used in studies investigating associations between somatic conditions and depression [22–24].

### Study population

**Fig. 1:**
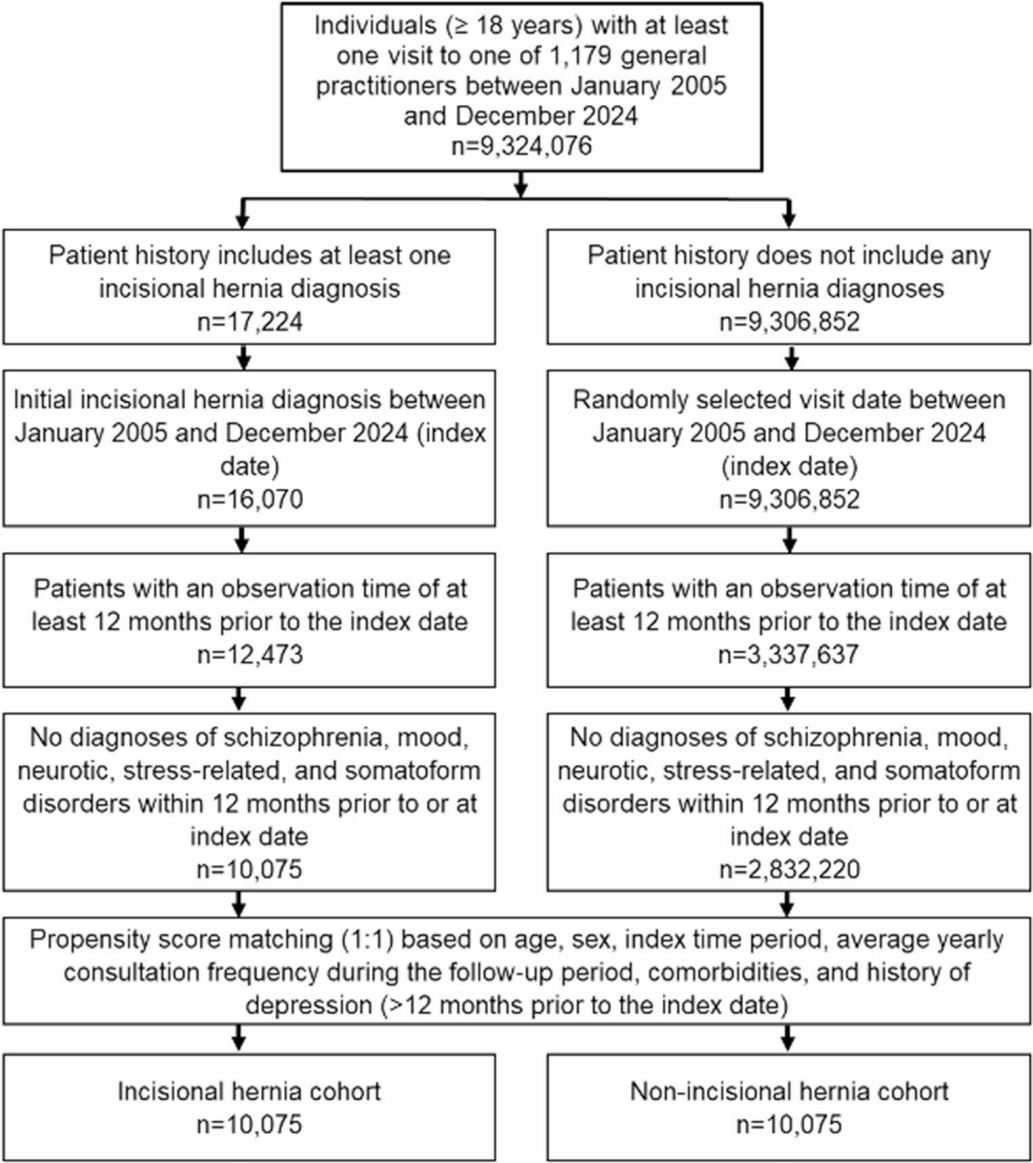
Selection of study patients

The study population consisted of adults aged 18 years or older with a first documented diagnosis of incisional hernia (ICD-10: K43.0, K43.1, K43.2) between January 2005 and December 2024 in 1,179 general practices (Fig. 1). The date of first documentation of incisional hernia within the study period was defined as the index date. An observation period of at least twelve months before the index date was required to ensure adequate baseline information. Individuals with schizophrenia, mood disorders, or neurotic, stress-related, and somatoform disorders recorded within twelve months prior to or on the index date were not included, as the aim was to analyse incident rather than prevalent depression.

To provide a comparable group of individuals without incisional hernia, persons fulfilling the same basic inclusion requirements were identified (Fig. 1). For these individuals, the index date was taken as a randomly selected visit within the study period. Both groups were then matched in a 1:1 nearest-neighbour propensity score procedure. Variables included in the matching were age, sex, index year, consultation frequency during follow-up, and a broad set of somatic diagnoses documented within five years prior to the index date, including obesity, hypertension, lipid metabolism disorders, diabetes mellitus, ischemic heart disease, cancer, ostomy status, back pain, headache, abdominal or pelvic pain, and any depression diagnosis occurring at least twelve months before the index date. After matching, balance across covariates was assessed using standardized mean differences, with values below 0.1 indicating sufficient comparability [25, 26].

The choice of matching variables reflected clinical and epidemiological considerations regarding potential confounding factors. Somatic comorbidities were included because they are often associated with elevated psychological burden and may independently influence the risk of depression. Pain-related conditions were matched because chronic or recurrent pain can contribute to mood deterioration and may increase the likelihood of receiving a depression diagnosis. Cancer and ostomy status were incorporated to account for differing levels of severe somatic burden and postoperative stress, which can also affect mental health. A history of depression documented at least twelve months before the index date was included in the matching process because earlier depressive episodes may increase a person’s general vulnerability to future depression. Incorporating this variable ensures that both groups have a comparable distribution of individuals with such a psychiatric background. This approach prevents systematic differences in long-term mental-health history from influencing the association between incisional hernia and subsequent depression. At the same time, using a twelve-month window guarantees that all depression diagnoses occurring after the index date are new events and not continuations of earlier episodes, allowing the investigation of whether a remote history of depression modifies the association between incisional hernia and later depression. Consultation frequency was added to minimize detection bias, as individuals who attend practices more frequently have a greater chance of having depression diagnosed simply due to more contact opportunities with healthcare professionals.

### Study outcomes and statistical analyses

The primary outcome of interest was a new diagnosis of depression (ICD-10: F32, F33) recorded within five years after the index date. For individuals in whom depression occurred, subsequent antidepressant prescriptions were also examined. Cumulative incidence functions for depression were estimated using Kaplan–Meier curves, displayed separately for different calendar periods. Associations between incisional hernia and the outcomes were quantified using univariable conditional Cox regression, and hazard ratios with 95% confidence intervals were reported. Because the matching procedure produced two groups with highly comparable distributions of all matched covariates and standardized mean differences consistently below the commonly accepted threshold, additional multivariable adjustment was deemed unnecessary. In such settings, univariable conditional Cox regression models provide a clear and appropriate analytical strategy. Additional adjustment after successful matching may introduce unnecessary complexity or instability and generally yields limited benefit when covariate balance has already been achieved. Analyses were conducted separately by age group, sex, and history of psychiatric disease. Because eight regression models were performed, statistical significance was defined as *p* < 0.006 after Bonferroni correction. All analyses were executed using SAS version 9.4 (SAS Institute, Cary, USA).

Because the Disease Analyzer database is based on routinely documented electronic medical records, variables were available only if documented by the treating physician. Missing data in the classical sense did not occur, as non-documentation was interpreted as absence of the diagnosis. No imputation procedures were applied.

## Results

### Basic characteristics of the study sample

A total of 10,075 individuals with incisional hernia and 10,075 individuals without incisional hernia were available for the final analyses (Fig. 1; Table 1). Among the 10,075 individuals with incisional hernia, 9,542 had incisional hernia without obstruction or gangrene, 492 had incisional hernia with obstruction without gangrene, and 41 had incisional hernia with gangrene. The mean age was 63.8 years in both groups, corresponding to 38.1% aged ≤ 60 years, 24.8% aged 61–70 years, 24.7% aged 71–80 years, and 12.2% aged > 80 years. Women represented 47.2% of individuals with incisional hernia and 46.8% of individuals without incisional hernia. The median number of physician visits per year during follow-up was 11–12 in both groups. A history of depression documented more than twelve months prior to the index date was documented in 17.0% of individuals with incisional hernia and 16.3% of individuals without incisional hernia.

**Table 1:** Baseline characteristics of the study sample (after propensity score matching)

Variable	Proportion among Individuals with Incisional hernia (%) *N* = 10,075	Proportion among individuals without Incisional hernia (%) *N* = 10,075	SMD
Age (Mean, SD)	63.8 (14.8)	63.8 (14.8)	0.000
Age ≤ 60	3,842 (38.1)	3,842 (38.1)	
Age 61–70	2,503 (24.8)	2,503 (24.8)	
Age 71–80	2,501 (24.7)	2,501 (24.7)	
Age > 80	1,229 (12.2)	1,229 (12.2)	
Women	4,757 (47.2)	4,717 (46.8)	0.003
Men	5,318 (52.8)	5,358 (53.8)	
2005–2008	538 (5.3)	664 (6.6)	0.004
2009–2012	1,021 (10.1)	1,052 (10.4)	
2013–2016	2,135 (21.2)	1,940 (19.3)	
2017–2020	2,863 (28.4)	2,700 (26.8)	
2021–2024	3,518 (34.9)	3,719 (36.9)	
Number of physician visits per year during the follow-up (Median, IQR)	11 (6)	12 (5)	-0.065
History of depression	1,709 (17.0)	1,642 (16.3)	-0.007
Diseases documented within five years prior to index date			
Obesity	1,937 (19.2)	1,941 (19.3)	0.000
Hypertension	6,009 (59.6)	6,080 (60.4)	0.007
Diabetes mellitus	2,041 (20.3)	1,974 (19.5)	-0.007
Lipid metabolism disorder	3,803 (37.8)	3,812 (37.8)	0.001
Ischemic heart diseases	2,084 (20.7)	2,023 (20.1)	-0.006
Cancer	2,913 (28.9)	2,887 (28.1)	-0.003
Ostomy status	650 (6.5)	466 (4.6)	0.018
Back pain	5,325 (52.9)	5,362 (53.2)	0.004
Headache	1,417 (14.1)	1,294 (12.8)	-0.012
Abdominal and pelvic pain	3,022 (30.0)	2,989 (29.7)	-0.003

Proportions of patients in % given, unless otherwise indicated. *SD *standard deviation

*SMD *standardized mean difference, *IQR *interquartile range

### Cumulative incidence of documented depression and antidepressant therapy

**Fig. 2:**
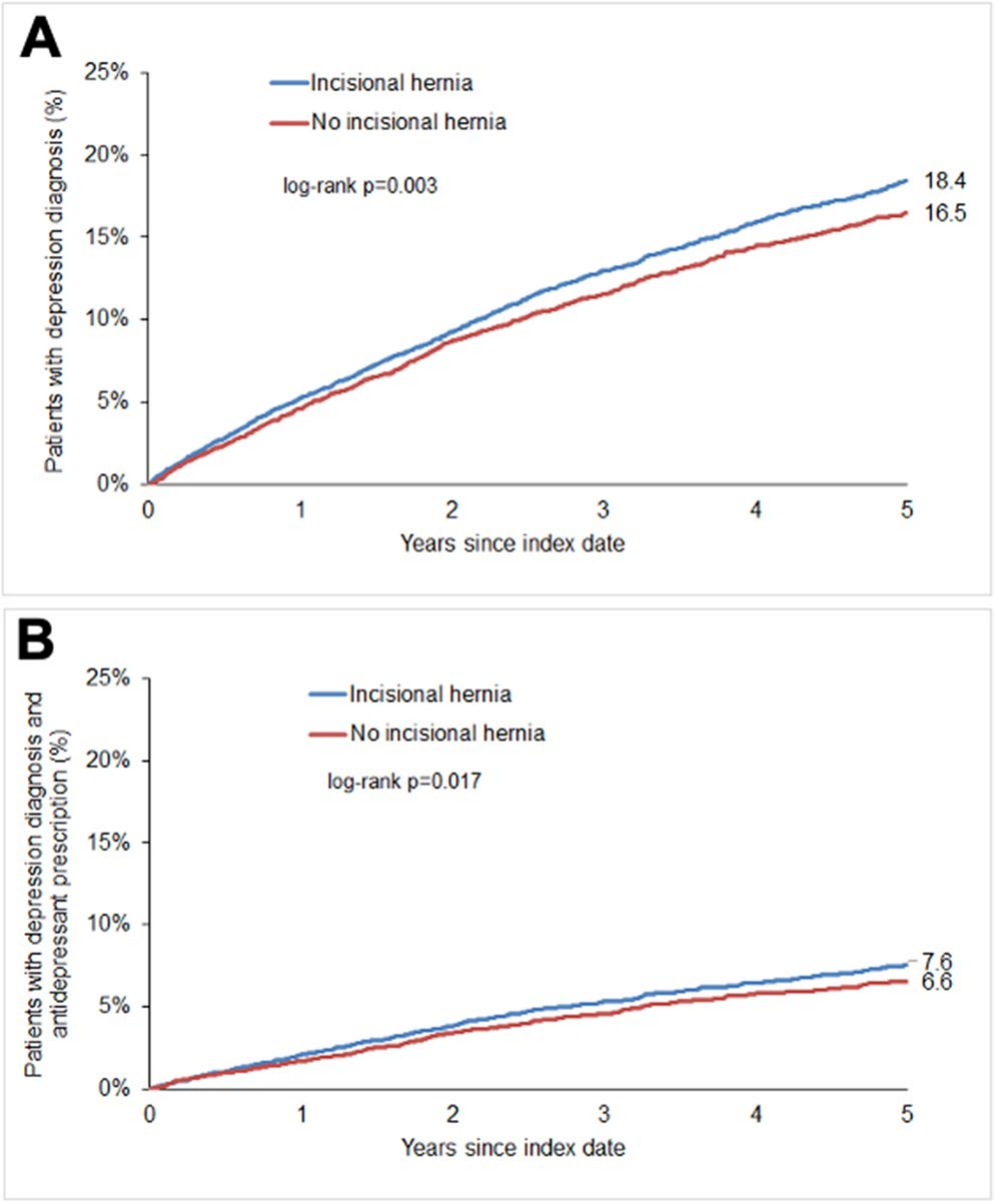
Cumulative incidence of psychiatric disorders in individuals with and without incisional hernia. (A) Depression and (B) Depression following by antidepressant prescription

Kaplan–Meier curves (Fig. 2) display the cumulative incidence of depression and subsequent antidepressant therapy in both cohorts. Over the five-year follow-up, the cumulative incidence of depression was 18.4% among individuals with incisional hernia and 16.5% among individuals without incisional hernia (*p* = 0.003). For antidepressant therapy, the corresponding 5-year cumulative incidence was 7.6% versus 6.6% (*p* = 0.017).

The incidence density of depression during follow-up was 37.8 cases per 1,000 person-years in individuals with incisional hernia and 35.7 cases per 1,000 person-years in individuals without incisionalhernia.

### Association of incisional hernia with subsequent depression and antidepressant therapy

The associations between incisional hernia and both outcomes are presented in Table 2. In the total sample, incisional hernia was associated with a small but statistically significant increase in risk of depression (HR: 1.12; 95% CI: 1.04–1.20). In the subgroup analyses, the association between incisional hernia and depression was significant only among women (HR: 1.15; 95% CI: 1.04–1.26) and among individuals without a history of depression (HR: 1.14; 95% CI: 1.04–1.25). In all other subgroups, the associations with depression were not significant.

**Table 2:** Association between incisional hernia and subsequent depression diagnosis and antidepressant therapy in patients followed in general practices in Germany (univariable Cox regression models)

Patients group	Depression diagnosis	Antidepressant prescription
	HR (95% CI)	HR (95% CI)
Total	1.12 (1.04–1.20)*	1.15 (1.03–1.29)
Age ≤ 60	1.15 (1.04–1.29)	1.23 (1.03–1.47)
Age 61–70	1.19 (1.01–1.38)	1.26 (0.98–1.64)
Age 71–80	1.00 (0.86–1.16)	0.96 (0.77–1.21)
Age > 80	1.06 (0.84–1.36)	1.12 (0.78–1.61)
Women	1.15 (1.04–1.26)*	1.16 (1.00-1.35)
Men	1.08 (0.96–1.21)	1.15 (0.95–1.39)
No history of depression	1.14 (1.04–1.25)*	1.23 (1.06–1.42)*
History of depression	1.09 (0.97–1.23)	1.03 (0.85–1.26)

*p < 0.006

The association with antidepressant therapy in the total sample (HR: 1.15; 95% CI: 1.03–1.29) did not meet the Bonferroni-adjusted significance threshold. Only among individuals without a history of depression, incisional hernia was associated with a modest but statistically significant increase in risk of receiving antidepressant therapy (HR: 1.23; 95% CI: 1.06–1.42). In all other subgroups, the associations with antidepressant therapy were not significant.

## Discussion

In this large, propensity score–matched cohort study, patients with newly diagnosed incisional hernia exhibited a statistically significant but small increase in risk of developing clinically documented depression during long-term follow-up compared with matched controls. Nearly one in five patients received a new diagnosis of depression within five years. The relative effect size observed in this study was modest. The HR of 1.12 indicates a small increase in relative risk, which should not be overinterpreted at the individual patient level. Given the observational design and the possibility of residual confounding, the findings should be interpreted cautiously. Nevertheless, incisional hernia is a common condition, and even small statistical associations may be relevant at the population level. Our findings therefore indicate a modest association between incisional hernia and subsequent depression diagnoses, suggesting that abdominal wall pathology may have psychosocial correlates beyond structural impairment alone.

It is important to emphasize that the present study was designed to examine an epidemiological association rather than to evaluate therapeutic effects of hernia repair. While previous research has primarily focused on QoL after surgical intervention, longitudinal data addressing whether incisional hernia itself is associated with subsequent clinically documented depression have been lacking. The current analysis therefore complements existing surgical outcome studies by providing population-based evidence from routine primary care.

Over recent years, qualitative and patient-centered investigations have increasingly challenged the reductionist view of incisional hernia as merely a mechanical defect. While Smith et al. highlighted themes of psychological vulnerability, altered identity, and social disconnection [10], subsequent qualitative work further delineated mental health burden and disturbances in body image as central components of abdominal wall disease [11, 12]. Rather than reiterating these descriptive findings, our data complement this literature by suggesting that such psychosocial strain may, in a subset of patients, translate into clinically recognized depressive morbidity within routine outpatient care.

The psychosocial dimension appears to be even broader. In a previously published qualitative analysis, Smith and colleagues investigated the impact of abdominal wall hernia on social and sexual relationships and reported significant disruption of interpersonal connection and intimacy [27]. Participants described social withdrawal driven by stigma, visible abdominal contour changes, pain, and reduced body confidence. Sexual relationships were frequently affected by discomfort, perceived loss of attractiveness, and fear of exacerbating symptoms. Emotional intimacy often persisted within supportive partnerships, yet loneliness and social disconnection were prominent. Notably, several patients described postoperative improvement in body confidence and social engagement, suggesting a potentially dynamic relationship between structural repair and psychosocial well-being.

These qualitative insights provide a critical interpretative framework for the present epidemiological findings. Social isolation, chronic pain, impaired intimacy, and negative body image are well-established contributors to depressive symptomatology [16, 28–30]. Depression is a leading cause of disability worldwide and affects hundreds of millions of individuals globally [15]. Within surgical populations, depressive symptoms have been linked to impaired postoperative recovery, increased pain perception, prolonged hospitalization, and reduced satisfaction with care [17, 18]. Against this background, the observed association between incisional hernia and depression diagnoses appears clinically plausible, although its magnitude was modest.

Psychological strain may further be amplified by treatment pathways. Patel et al. explored in a recent qualitative study the impact of delayed or deferred ventral hernia repair in patients undergoing surgical optimization [31]. Beyond physical symptoms, patients reported fear of progression, uncertainty regarding the future, frustration with perceived loss of autonomy, and distress associated with feeling “not taken seriously”. Emotional distress was frequently compounded by the experience of prolonged waiting and conditional access to surgery. These findings highlight that both disease presence and treatment pathways may contribute to psychological vulnerability. From a clinical perspective, this underscores the importance of transparent communication and shared decision-making when surgical delay is considered. Our population-level data suggest that such psychosocial stressors may be reflected in routine psychiatric diagnoses.

Importantly, evidence from randomized and observational studies indicates that surgical repair can substantially improve HRQoL. In the PROLOVE randomized controlled trial, Rogmark and colleagues reported significant improvement in multiple SF-36 domains one year after incisional hernia repair, with postoperative scores approaching population norms [14]. Similarly, a recent systematic review demonstrated moderate to large improvements across validated QoL instruments following complex incisional hernia repair [13]. Although mental composite scores did not uniformly improve across all cohorts, reductions in pain, movement limitation, and abdominal wall complaints were consistent. These findings suggest that structural restoration may alleviate some psychosocial stressors identified in qualitative research.

However, the quality and consistency of long-term patient-reported outcome data in ventral and incisional hernia surgery remain variable. A recent systematic review evaluating long-term patient-reported outcomes after ventral hernia mesh repair highlighted substantial heterogeneity in measurement instruments and overall limited methodological quality of the available evidence [32]. Importantly, structured longitudinal assessment of mental health trajectories was rarely performed. This underscores that, despite increasing attention to HRQoL, robust epidemiological data on clinically diagnosed psychiatric outcomes have been lacking.

At the same time, psychological vulnerability appears to be prevalent even before operative intervention. In this context, a recently published prospective pilot study reported that nearly two-thirds of patients scheduled for complex abdominal wall reconstruction screened positive for at least one psychological risk factor, including anxiety, depressive symptoms, post-traumatic stress disorder, or reduced self-efficacy [33]. Moreover, these factors were associated with prolonged postoperative pain. This observation is particularly relevant when interpreting the present findings: psychological comorbidity may represent both a consequence of the disease process and a predisposing factor influencing recovery. The relationship between hernia pathology and depression is therefore likely bidirectional and dynamic.

The way in which psychological burden is measured warrants attention. Current HRQoL instruments may incompletely capture the multidimensional psychosocial impact of abdominal wall pathology, particularly domains such as body image, social integration, and emotional distress. The development of the Abdominal Hernia-Q reflects an effort to systematically incorporate domains that patients consider relevant, including symptom burden, physical limitation, body image, and social functioning [34]. The inclusion of psychosocial dimensions within this hernia-specific instrument highlights that QoL impairment extends beyond structural recurrence or technical outcomes.

In response to these limitations, Abbas et al. recently proposed an artificial intelligence–based framework aimed at enhancing the interpretative utility of HRQoL assessment tools in abdominal wall surgery [35]. Our findings lend quantitative support to this argument. If incisional hernia is associated with increased long-term depression risk, then refinement of patient-reported outcome assessment is not merely methodological but clinically relevant. More nuanced measurement tools may facilitate earlier identification of vulnerable patients and enable tailored perioperative support strategies.

Several mechanisms may plausibly contribute to the observed association between incisional hernia and incident depression. Persistent pain and activity-related discomfort may function as sustained affective stressors, as chronic pain has consistently been linked to depressive symptomatology and altered central pain processing [36, 37]. Functional restriction, including reduced mobility and lifting capacity, may limit participation in occupational and recreational activities and thereby undermine autonomy; disability has been shown to correlate bidirectionally with depressive symptoms across medical populations [38]. In patients with incisional hernia, visible abdominal contour changes and altered body integrity may further impair self-perception and social confidence; body image disturbance has been prospectively documented in this population and shown to correlate with reduced QoL [12, 39]. Finally, uncertainty regarding disease progression or timing of surgical repair may contribute to anticipatory anxiety and emotional strain, particularly when operative management is delayed [31]. These mechanisms are unlikely to operate independently and may interact to amplify psychosocial burden. However, these factors were not directly measured in the present dataset and are discussed here as plausible explanatory frameworks derived from prior literature rather than empirically assessed variables.

This study has several strengths, including a large representative cohort, robust propensity score matching, and extended longitudinal follow-up. By excluding patients with recent psychiatric diagnoses, we strengthened the assessment of incident depression rather than prevalent disease.

However, we also have to acknowledge several limitations that should be considered when interpreting the findings. First, the IQVIA Disease Analyzer database contains diagnoses and prescriptions documented by general practitioners but does not provide granular information regarding hernia size, symptom severity, cosmetic impact, or chronic postoperative pain. Consequently, we were unable to assess hernia size, defect width, grading, symptom burden, or morphological characteristics.

Second, although body mass index is recorded in a small subset of practices, it is not available consistently enough to be used reliably in propensity score matching. For this reason, we relied on physician-documented obesity diagnoses, which are uniformly available. Other important determinants of depression, such as socioeconomic status, employment, smoking, alcohol use, and psychosocial stressors, are not captured in the database. Psychiatric medication history is only available when prescriptions are issued by general practitioners; medications prescribed by psychiatrists or inpatient facilities are not included. Consequently, although propensity score matching improved balance for measured covariates, it cannot eliminate residual confounding due to unmeasured variables.

Third, the database does not include information on prior abdominal operations that may have resulted in the incisional hernia. Surgical history, including type, timing, indication, or complexity of the index procedure, is not available. As a result, we cannot disentangle the psychological impact of the preceding surgery from that of the hernia itself.

Fourth, information on operative management of the hernia is unavailable. The Disease Analyzer database does not contain procedure codes or hospital data, and therefore we cannot determine which patients underwent hernia repair, nor can we assess type of hernia repair, postoperative outcomes, recurrence, or changes in depression risk following repair. Subgroup analyses comparing operated versus non-operated patients or recurrent versus non-recurrent hernias were therefore not feasible.

Fifth, depression diagnoses were based on routine ICD-10 coding in outpatient practice rather than standardized psychiatric interviews. While misclassification is possible, such nondifferential misclassification would likely bias results toward the null.

Finally, the database does not capture pain severity, functional limitation, or body image concerns. Mechanisms discussed in the manuscript, such as chronic pain, reduced mobility, or body image disturbance, are therefore based on prior literature and cannot be directly evaluated in this dataset.

Finally, the present study was not intended to assess whether surgical repair of incisional hernia reduces depressive symptoms or alters long-term psychiatric trajectories. Such analyses would require linkage with procedural and hospital-based data. The objective was exclusively to determine whether a statistically detectable association exists between the diagnosis of incisional hernia and subsequent depression in routine outpatient care. This distinction is important when interpreting the scope and implications of the findings.

In summary, this population-based analysis demonstrates that incisional hernia is associated with a small but statistically significant increase in the risk of clinically diagnosed depression in routine care. When interpreted alongside contemporary qualitative, interventional, and methodological research, these findings support a multidimensional understanding of abdominal wall disease. Recognition of potential psychosocial comorbidity may inform future research and clinical awareness, although the modest magnitude of association observed here does not justify changes in clinical management based solely on these findings.

## Supplementary Information

Below is the link to the electronic supplementary material.

Supplementary Material 1

## Data Availability

The data used in this study are derived from the IQVIA Disease Analyzer database and are subject to licensing restrictions. Data are available from IQVIA upon reasonable request and with permission of the data provider.
